# Supplementary material for: Effect of SARS-CoV-2 proteins on vascular permeability
Source: eLife. 2021 Oct 25;10:e69314. doi: 10.7554/eLife.69314 (PMC8545399; doi:10.7554/eLife.69314)
Supplement: Supplementary file 1. — (A) Documented change by severe acute respiratory syndrome (SARS)-Cov-2. (B) Significant target for each viral protein. [file elife-69314-supp1.docx]

**Supplementary Information**

**Effect of SARS-CoV-2 proteins on vascular permeability**

Rossana Rauti^1*^, Meishar Shahoha^2,3*^, Yael Leichtmann-Bardoogo^1 *^, Rami Nasser^4^, Eyal Paz^2,3^, Rina Tamir^1^, Victoria Miller^1^, Tal Babich^1,2^, Kfir Shaked ^1,2^, Avner Ehrlich^5^, Konstantinos Ioannidis^5^, Yaakov Nahmias ^5^, Roded Sharan^4^, Uri Ashery ^2,3,6#^, and Ben M. Maoz^1,3,6#^

^1^Department of Biomedical Engineering, Tel Aviv University, Israel

^2^School of  Neurobiology, Biochemistry and Biophysics, The George S. Wise Faculty of Life Sciences, Tel Aviv University, Tel Aviv, Israel

^3^Sagol School of Neuroscience, Tel Aviv University, Tel Aviv, Israel

^4^Blavatnik School of Computer Science, Tel Aviv University, Tel Aviv, Israel

^5^Hebrew University of Jerusalem - Grass Center for Bioengineering

^6^The Center for Nanoscience and Nanotechnology, Tel Aviv University, Israel

^*^ These authors are equal contributors

# Shared corresponding authors

Corresponding author

Uri Ashery, mail: uriashery@gmail.com

Ben M. Maoz, mail: bmaoz@tauex.tau.ac.il

**Supplementary File 1A. Documented change by SARS-Cov-2.**

| **Cell type** | **Function** | **Gene** | **Entrez Gene** | **Identified significant SARS-Cov-19 proteins** | **Distance** |
| --- | --- | --- | --- | --- | --- |
| Dendritic cells (T cell proliferation) | Impaired dendritic cells, induces T-cell proliferation and cytokine production and signal for activation of the T-cell | CD80^1^ | 941 | [e; m; spike; nsp10; nsp11; nsp15; nsp7; orf3a; orf6; orf7a; orf8; orf9b; orf9c] | 2 |
|  |  | CD86^1^ | 942 | [nsp10; nsp7] | 2 |
|  |  | INFα^1^ | 3439 |  | 2 |
|  |  | INF β^1^ | 3456 |  | 2 |
| T cell | Impaired T cell maturation and activation, reduced proliferation | INF λ^1-4^ | 3458 |  | 2 |
|  |  | TNFα^1,3,4^ | 7124 | [nsp12; orf8] | 1 |
|  | Production of pro-inflammatory cytokines | IL-2^2,3,4^ | 3558 | [m; spike; nsp11; nsp12; nsp2; nsp7; orf10; orf8; orf9c] | 2 |
|  |  | IL-10^2,3,4^ | 3586 | [spike; orf8; orf9c] | 2 |
|  |  | IL-7^2^ | 3574 | [m; orf10; orf9c] | 2 |
|  |  | IL-6^2,4^ | 3569 | [nsp11; nsp14; nsp5; nsp6; nsp9; orf10; orf8; orf9b] | 2 |
|  |  | IL-1β^2^ | 3553 | [orf8] | 2 |
|  |  | INF-ɑ^2^ | 3439 |  | 2 |
|  |  | MOP-1^2^ |  |  |  |
|  |  | IP-10^2^ | 3627 |  | 2 |
|  | reduced proliferation | CTLA-4^3^ | 1493 | [nsp10; nsp15; nsp7; orf9b] | 1 |
|  |  | PD-1^1,3^ | 5133 | [m; spike; nsp15; nsp4; orf6; orf9c] | 1 |
|  |  | Ki67^3^ | 4288 | [n; nsp5; nsp7; nsp8; orf3a; orf3b; orf7a] | 1 |
|  |  | IL-5^3^ | 3567 | [spike; nsp10; orf8] | 2 |
|  |  | IL-13^3^ | 3596 | [m; spike; nsp10; nsp11; nsp12; nsp13; nsp2; nsp4; nsp6; nsp7; orf3a; orf3b; orf7a; orf8; orf9c] | 2 |
|  |  | IL-9^3,4^ | 3578 |  |  |
|  |  | IL-22^3^ | 50616 | [spike; orf9c] | 3 |
| NK | Decreased **IFN-γ production** by NK cells | INFλ^5^ | 3458 |  | 2 |
| Myeloid cells | systemic inflammation may impair the myeloid cells functionality | HLA-DR^6^ |  |  |  |
|  |  | CD64^6^ | 2209 |  | 2 |
|  |  | CD66b^6^ | 1088 | [m; spike; nsp10] | 2 |
|  |  | CRP^6^ | 1401 | [orf8] | 1 |
|  |  | Ferritin l^6^ | 2512 |  | 1 |
|  |  | ferritin h^6^ | 2495 | [nsp4] | 1 |
|  |  | D-dimer^6^ |  |  |  |
|  |  | ﬁbrinogen^7^ | |  |  |
|  |  | LDH a^6^ | 3939 | [m; nsp11; nsp14; nsp4; nsp7; orf10; orf3b] | 1 |
|  |  | IL6^6^ | 3569 | [nsp11; nsp14; nsp5; nsp6; nsp9; orf10; orf8; orf9b] | 2 |
| Lymphocyte | severe lymphocyte apoptosis | FAS^7^ | 355 | [nsp11; nsp2; nsp4; nsp7; orf3a; orf6; orf7a; orf9c] | 1 |
| Monocyte and macrophage | Activation and transcription of proinflammatory genes | ACE2^4^ | 59272 | [e; nsp10; nsp12; nsp15; nsp2; nsp4; nsp6; orf10; orf7a; orf8; orf9b; orf9c] | 1 |
|  |  | IL6^4^ | 3569 | [nsp11; nsp14; nsp5; nsp6; nsp9; orf10; orf8; orf9b] | 2 |
|  |  | IL-10^4^ | 3586 | [spike; orf8; orf9c] | 2 |
|  |  | TNFα^4^ | 7124 | [nsp12; orf8] | 1 |
|  |  | ITGAM  cd1b^4^ | 3684 | [nsp11] | 2 |
|  |  | CD14^4^ | 929 | [nsp5] | 2 |
|  |  | FCGR3Acd16^4^ | 2214 | [nsp10] | 2 |
|  |  | CD68^4^ | 968 | [m; orf6; orf7a] | 1 |
|  |  | CD80^4^ | 941 | [e; m; spike; nsp10; nsp11; nsp15; nsp7; orf3a; orf6; orf7a; orf8; orf9b; orf9c] | 2 |
|  |  | CD163^4^ | 9332 | [n] | 1 |
|  |  | MRC1 cd206^4^ | 4360 | [orf8] | 2 |
|  |  | PDCD1  PD1^4^ | 5133 |  | 1 |
| PB mononuclear cells | Activation of proinflammatory genes | IL1B^4^ | 3553 | [orf8] | 2 |
|  |  | CXCL2^4^ | 2920 | [nsp4; nsp9] | 2 |
|  |  | CXCL8^4^ | 3576 |  | 2 |
| PBMC | Activation of proinflammatory genes | GDF11^8^ | 10220 | [nsp1; nsp5_c145a; orf8] | 1 |
|  |  | NOG^8^ | 9241 | [spike; nsp5_c145a; orf3a; orf8; orf9c] | 2 |
|  |  | IL10^8^ | 3586 | [spike; orf8; orf9c] | 2 |
|  |  | CXCL10^8^ | 3627 | nsp4 | 2 |
|  |  | TNFSF10^8^ | 8743 | [spike; nsp11; nsp12; nsp14; nsp15; orf10; orf3a; orf9b] | 1 |
|  |  | NRG1^8^ | 3084 | [spike; nsp11; nsp15; nsp4; nsp6; orf3a; orf6; orf7a; orf8; orf9c] | 1 |
|  |  | TIMP1^8^ | 7076 | [nsp10; nsp6; nsp9; orf8] | 2 |
|  |  | C5^8^ | 727 | [nsp11; nsp4; orf9c] | 2 |
|  |  | IL18^8^ | 3606 | [nsp2; orf7a] | 2 |
|  |  | AREG^8^ | 374 | [e; nsp12; nsp6] | 2 |
|  |  | CCL4^8^ | 6351 | [spike; nsp4; orf9c] | 1 |
|  |  | CMTM2^8^ | 146225 | [orf3a; orf9c] | 1 |
|  |  | CXCL8^8^ | 3576 |  | 2 |
|  |  | IFNλ^8^ | 3458 | [spike; nsp11; nsp13; nsp4; nsp7; orf9c] | 2 |
| Lymphocytes | SARS-CoV-2 infection may cause lymphocyte apoptosis | CTSL^8^ | 1514 | [nsp14] | 1 |
|  |  | CTSB^8^ | 1508 | [m; nsp11; nsp4; nsp5_c145a; nsp6; orf9b] | 1 |
|  |  | DDIT4^8^ | 54541 | [orf9b] | 2 |
|  |  | RRAS^8^ | 6237 | [m; nsp2] | 1 |
|  |  | CTSD^8^ | 1509 | [m; nsp11; nsp14; nsp2; nsp4; nsp5_c145a; nsp6; nsp7; orf10; orf6; orf8] | 1 |
|  |  | BIRC5^8^ | 332 | [nsp14; nsp15] | 1 |
|  |  | TNFSF10^8^ | 8743 | [spike; nsp11; nsp12; nsp14; nsp15; orf10; orf3a; orf9b] | 1 |
|  |  | CTSZ^8^ | 1522 |  | 1 |
|  |  | NTRK1^8^ | 4914 |  | 1 |
|  |  | IGFBP3^8^ | 3486 | [nsp1; nsp14; nsp15; nsp4; orf8; orf9b] | 1 |
|  |  | CCNB1^8^ | 891 | [nsp1; nsp11; nsp13] | 1 |
|  |  | RRM2^8^ | 6241 | [nsp1; orf6] | 2 |
|  |  | CCNB2^8^ | 9133 | [nsp1; nsp10; nsp11; nsp13; nsp14; nsp15; nsp2; orf8; orf9b] | 1 |
|  |  | GTSE1^8^ | 51512 | [nsp10; nsp12; nsp13] | 1 |
|  |  | CDK1^8^ | 983 | [nsp1; orf3b] | 1 |
|  |  | STEAP3^8^ | 55240 | [spike; nsp4; nsp6; orf3a; orf7a] | 2 |
|  |  | TP53I3^8^ | 9540 |  | 2 |
| BALF | Activation of proinflammatory cytokines | CXCL2^8^ | 2920 | [nsp4; nsp9] | 2 |
|  |  | TGFB2^8^ | 7042 | [e; nsp4; nsp9; orf6; orf8; orf9b] | 2 |
|  |  | CCL3L1^8^ | 6349 | [spike; nsp4] | 2 |
|  |  | CXCL6^8^ | 6372 | [nsp4; nsp9] | 2 |
|  |  | CCL8^8^ | 6355 | [nsp4; nsp9] | 2 |
|  |  | SCGB3A1^8^ | 92304 |  | 2 |
|  |  | TNFSF10^8^ | 8743 | [spike; nsp11; nsp12; nsp14; nsp15; orf10; orf3a; orf9b] | 1 |
|  |  | CCL2^8,9^ | 6347 | [nsp4; nsp9] | 2 |
|  |  | CXCL1^8^ | 2919 | [orf10] | 1 |
|  |  | IL33^8^ | 90865 | [e; nsp10; nsp5; orf8; orf9b] | 2 |
|  |  | GRN^8^ | 2896 | [nsp1; nsp11] | 1 |
|  |  | CXCL16^8^ | 58191 |  | 1 |
| Immune cells of BALF | higher levels of inflammatory cytokines in lung macrophages from patients with severe COVID-19 infection. | IL-8^9^ | 3576 |  | 2 |
|  |  | IL-6^9^ | 3569 | [nsp11; nsp14; nsp5; nsp6; nsp9; orf10; orf8; orf9b] | 2 |
|  |  | IL-1β^9^ | 3553 | [orf8] | 2 |
|  |  | TNFα^9^ | 7124 | [nsp12; orf8] | 1 |
|  |  | CCL3^9^ | 6348 | [m; nsp4; nsp7; orf9c] | 1 |
|  |  | CCL4^9^ | 6351 | [spike; nsp4; orf9c] | 1 |
|  |  | CCL7^9^ | 6354 | [spike; orf8] | 1 |
|  |  | CXCL9^9^ | 4283 | [orf9c] | 1 |
|  |  | CXCL10^9^ | 3627 | [nsp4] | 2 |
|  |  | CXCL11^9^ | 6373 | [nsp4] | 2 |
| Primary lung epithelial cells | glycolysis | HK2*^10^ | 3099 | [nsp1; nsp11; nsp6; orf6; orf8; orf9b] | 1 |
|  | glycolysis | GPI^10^ | 2821 | [nsp11; nsp14; orf3] | 1 |
|  | glycolysis | PFKP^10^ | 5214 | [nsp11; nsp14; nsp15; nsp5_c145a; orf7a] | 1 |
|  | glycolysis | ENO2*^10^ | 2026 | [nsp11; nsp14; nsp15; nsp4; nsp5_c145a; nsp7; orf10] | 1 |
|  | glycolysis | PKM*^10^ | 5315 | [nsp11; nsp5; nsp7; orf7a; orf9b] | 1 |
|  | glycolysis | LDHA^10^ | 3939 | [m; nsp11; nsp14; nsp4; nsp7; orf10; orf3b] | 1 |
|  | glycolysis | ALDOA^10^ | 226 | [nsp11; nsp5_c145a; orf3b; orf6] | 1 |
|  | glycolysis | PGK1^10^ | 5230 | [nsp11; nsp14; nsp5_c145a; orf3b; orf6] | 1 |
|  | glycolysis | BPGM*^10^ | 669 | [nsp15] | 2 |
|  | Pentose-Phosphate | PGD^10^ | 5226 | [m; nsp11; nsp14; nsp15; nsp4; nsp5_c145a; nsp9; orf3b; orf6; orf9b] | 1 |
|  | Pentose-Phosphate | PGLS^10^ | 25796 | [nsp10; nsp11; nsp15; nsp4; nsp5_c145a] | 1 |
|  | Pentose-Phosphate | RBKS^10^ | 64080 | [nsp11; nsp13; nsp15; nsp4; orf3a] | 2 |
|  | mitochondria | DNAJC15*^10^ | 29103 | [nsp7] | 2 |
|  | mitochondria | ATP1A1*^10^ | 476 | [m; nsp11; nsp6; nsp7; orf10; orf3b; orf6] | 1 |
|  | mitochondria | UQCRB^10^ | 7381 | [m; spike; nsp11; nsp2; nsp4; nsp5_c145a; nsp6; nsp7; orf3b; orf6] | 1 |
|  | mitochondria | IDH1*^10^ | 3417 | [nsp10; nsp11; nsp14; nsp15; nsp4; nsp5_c145a; orf3b; orf6] | 1 |
|  | mitochondria | CDK1*^10^ | 983 | [nsp1; orf3b] | 1 |
|  | palmitoylation | ZDHHC14^10^ | 79683 |  | 2 |
|  | palmitoylation | IFITM1*^10^ | 8519 |  | 2 |
|  | palmitoylation | IFITM3*^10^ | 10410 | [nsp6; orf3b] | 1 |
|  | lipogenesis | ACAA1^10^ | 30 | [spike; nsp11; nsp12; nsp14; nsp15; nsp5_c145a; nsp6; orf3b] | 1 |
|  | lipogenesis | HMGCS1*^10^ | 3158 | [nsp13] | 1 |
|  | lipogenesis | ACAT2^10^ | 39 | [n; nsp10; nsp11; nsp14; nsp15; nsp4; nsp5_c145a; nsp6; orf3b; orf6; orf7a; orf9b] | 1 |
|  | lipogenesis | BCKDK^10^ | 10295 | [nsp14; nsp6; orf7a] | 0 |
|  | lipogenesis | MVD^10^ | 4597 | [nsp10; nsp11; nsp14; nsp2; nsp4; orf3b; orf9b] | 2 |
|  | lipogenesis | FDFT1^10^ | 2222 | [nsp6; nsp7; orf3a; orf3b] | 1 |
|  | lipogenesis | SQLE*^10^ | 6713 | [m; nsp4; nsp6; orf9c] | 2 |
|  | lipogenesis | DGAT1^10^ | 8694 | [m; spike; nsp6; orf3a; orf9c] | 2 |
|  | lipogenesis | MTTP^10^ | 4547 | [nsp7; orf8] | 2 |
|  | oxidative stress | NFKB1*^10^ | 4790 | [nsp13; nsp5] | 1 |
|  | oxidative stress | JUN^10^ | 3725 | [e; m; n; nsp1; nsp10; nsp12; nsp13; nsp15; nsp4; nsp5; nsp7; nsp8; nsp9; orf10; orf3b; orf6; orf7a; orf9b] | 1 |
|  | oxidative stress | FOXO1*^10^ | 2308 | [nsp1; nsp11; nsp14; nsp15; nsp2; nsp5] | 1 |
|  | oxidative stress | GCLC*^10^ | 2729 | [nsp14; nsp15; nsp9; orf9b] | 1 |
|  | oxidative stress | HMOX2*^10^ | 3163 | [m; nsp2; nsp4; nsp6; nsp7; orf3b; orf9c] | 1 |
|  | oxidative stress | IL6*^10^ | 3569 | [nsp11; nsp14; nsp5; nsp6; nsp9; orf10; orf8; orf9b] | 2 |
|  | oxidative stress | SOD2^10^ | 6648 | [m; nsp11; nsp14; nsp15; nsp5_c145a; nsp6; orf3b; orf6; orf8] | 1 |
|  | ER stress | ERN1^10^ | 2081 | [nsp10; nsp11; nsp4; nsp5_c145a; nsp6; nsp9; orf3a; orf8; orf9b; orf9c] | 1 |
|  | ER stress | EIF2AK2^10^ | 5610 | [n] | 1 |
|  | ER stress | ATF4^10^ | 468 | [nsp9] | 1 |
|  | ER stress | CEBPB^10^ | 1051 |  | 2 |
|  | ER stress | DNAJB9^10^ | 4189 | [nsp11; orf7a; orf9c] | 2 |
| Cardiomyocytes | cardiac muscle tissue development  regulation of viral process  actin filament organization  proteosomal protein catabolic process  cellular respiration  regulation of lymphocyte activation  sensory perception of smell  pos. reg. of receptor sig.pathway via JAK-STAT | ATP2A2^11^ | 488 | [m; nsp2; nsp6; nsp7; orf3b; orf6; orf7a; orf9c] | 1 |
|  |  | ENO3^11^ | 2027 | [nsp11; nsp13; nsp4; orf3b; orf6; orf9b] | 1 |
|  |  | ACTB^11^ | 60 | [m; spike; nsp5; nsp7; orf10; orf3b; orf9c] | 1 |
|  |  | TPM2^11^ | 7169 |  | 1 |
|  |  | TPM4^11^ | 7171 | [nsp11; nsp6; orf3b] | 1 |
|  |  | CALM3^11^ | 808 | [nsp11; nsp13; nsp7] | 1 |
|  |  | TPM1^11^ | 7168 | [spike; nsp11; nsp13; orf3b] | 1 |
|  |  | CALM2^11^ | 805 | [nsp11; nsp13; nsp7] | 1 |
|  |  | MYL9^11^ | 10398 | [e] | 1 |
|  |  | MYL6^11^ | 4637 | [nsp11; nsp13; nsp15; orf10; orf3b] | 1 |
|  |  | TNNC1^11^ | 7134 | [orf9b] | 2 |
|  |  | CKM^11^ | 1158 | [e; orf10; orf6; orf9b] | 1 |
|  |  | MYL7^11^ | 58498 |  | 2 |
|  |  | MYH11^11^ | 4629 | [nsp11; nsp14; orf3b] | 1 |
|  |  | MYLK^11^ | 4638 | [e] | 2 |
|  |  | MYL4^11^ | 4635 | [n; nsp10] | 2 |
|  |  | TNNI1^11^ | 7135 |  | 2 |
|  |  | TPM3^11^ | 7170 | [nsp11; nsp6; orf6] | 1 |
|  |  | MYH6^11^ | 4624 | [nsp14; orf9c] | 1 |
|  |  | TNNI3^11^ | 7137 | [nsp10; nsp11; nsp13; nsp15; orf3a; orf9b] | 1 |
|  |  | CAPZB^11^ | 832 | [nsp11; nsp5_c145a; orf9b] | 1 |
|  |  | ACTC1^11^ | 70 | [nsp1] | 1 |
|  |  | MYOM2^11^ | 9172 |  | 2 |
|  |  | TMOD1^11^ | 7111 | [spike; orf6] | 1 |
|  |  | TTN^11^ | 7273 | [orf3a] | 1 |
|  |  | MYH8^11^ | 4626 | [e] | 1 |
|  |  | MYO1G^11^ | 64005 | [nsp15; nsp2; nsp6; nsp7; orf3b] | 1 |
|  |  | TRIM63^11^ | 84676 |  | 1 |
|  |  | TNNT3^11^ | 7140 | [nsp10; nsp13] | 1 |
|  |  | NEB^11^ | 4703 | [e; nsp1] | 2 |
|  |  | MYL3^11^ | 4634 |  | 2 |
|  |  | OBSCN^11^ | 84033 | [nsp6; nsp9; orf3a] | 1 |
|  |  | MYH2^11^ | 4620 | [spike] | 1 |
|  |  | MYO7A^11^ | 4647 | [orf3b] | 2 |
|  |  | MYO3A^11^ | 53904 |  | 2 |
|  |  | MYH7^11^ | 4625 |  | 1 |
|  |  | MYH15^11^ | 22989 | [nsp7; orf8; orf9c] | 2 |
|  |  | MYO3B^11^ | 140469 | [m; nsp10; nsp5_c145a] | 1 |
|  |  | MYO1H^11^ | 283446 |  | 1 |
|  |  | MYO15A^11^ | 51168 | [nsp13] | 2 |
|  |  | MYLK2^11^ | 85366 | [nsp1; nsp4; orf7a] | 2 |
|  |  | MYO7B^11^ | 4648 | [m] | 2 |
|  |  | MYO1A^11^ | 4640 | [nsp9] | 1 |
|  |  | MYH4^11^ | 4622 |  | 1 |
|  |  | MYH1^11^ | 4619 |  | 1 |
|  |  | MYH13^11^ | 8735 | [nsp5] | 1 |
|  |  | MYO1F^11^ | 4542 | [orf8] | 1 |
| Human bronchial epithelial cell line (16HBE) | Activation of proinflammatory cytokines | IKKβ, ^12^ | 3551 | [nsp12; nsp13; orf9b] | 1 |
|  |  | IFNλ^12^ | 3458 | [spike; nsp11; nsp13; nsp4; nsp7; orf9c] | 2 |
|  |  | TNF‐α^12^ | 7124 | [nsp12; orf8] | 1 |
|  |  | LTa3^12^ |  |  |  |
|  |  | GMCSF^12^ |  |  |  |
|  |  | IL‐1β^12^ | 3553 | [orf8] | 2 |
|  |  | IL‐2^12^ | 3558 | [m; spike; nsp11; nsp12; nsp2; nsp7; orf10; orf8; orf9c] | 2 |
|  |  | IL‐6^12^ | 3569 | [nsp11; nsp14; nsp5; nsp6; nsp9; orf10; orf8; orf9b] | 2 |
|  |  | IL‐8^12^ | 3576 |  | 2 |
|  |  | IL‐12^12^ | 3593 | [nsp11; nsp15; nsp8; orf8] | 1 |
|  |  | IL‐13^12^ | 3596 | [m; spike; nsp10; nsp11; nsp12; nsp13; nsp2; nsp4; nsp6; nsp7; orf3a; orf3b; orf7a; orf8; orf9c] | 2 |
|  |  | IL‐33^12^ | 90865 | [e; nsp10; nsp5; orf8; orf9b] | 2 |
|  |  | IL‐4^12^ | 3565 | [m; spike; nsp10; nsp2; nsp4; nsp6; nsp7; orf10; orf8; orf9b; orf9c] | 2 |
|  |  | IL‐5^12^ | 3567 | [spike; nsp10; orf8] | 2 |
|  |  | IL‐10^12^ | 3586 | [spike; orf8; orf9c] | 2 |
|  |  | IL‐17^12^ | 3605 | [spike; nsp12; orf8; orf9c] | 1 |
|  |  | IL‐22^12^ | 50616 | [spike; orf9c] | 3 |
| Neuronal cells | altered distribution of Tau | [Tau](https://www.ncbi.nlm.nih.gov/gene/4137)^13^ | 4137 | [n; nsp1; nsp10; nsp13; nsp7; nsp8; orf10; orf7a; orf9b] | 1 |
|  | apoptosis of neuronal cells | Caspase 3^13^ | 836 | [nsp11; nsp12; nsp13; nsp14; nsp15; nsp4; nsp5_c145a; orf3a] | 1 |
|  |  | UNC13A | 23025 | [n; orf6] | 1 |
|  |  | RAB27A | 5873 | [nsp7] | 2 |
|  |  | RAB3A | 5864 | [nsp1; nsp10; nsp11; nsp15; nsp7; orf6; orf9c] | 1 |
|  |  | RIMS1 | 22999 | [nsp7; orf9b] | 2 |
|  |  | RPH3A | 22895 | [nsp7] | 1 |
|  |  | SNAP25 | 6616 | [spike; nsp10; nsp11; nsp15; nsp2; nsp4; nsp6; orf3a; orf9b] | 2 |
|  |  | SNCA | 6622 | [nsp11; nsp5_c145a; nsp6; nsp7; orf3b; orf8] | 1 |
|  |  | STX1A | 6804 | [orf3a] | 1 |
|  |  | STXBP1 | 6812 | [nsp10; nsp11; nsp15; nsp2; nsp4; orf3a] | 1 |
|  |  | STXBP5 | 134957 | [e; spike; nsp2; nsp6; orf3a; orf9b; orf9c] | 2 |
|  |  | Syp | 6855 | [nsp11] | 2 |
| Proximal tubule cells | decreased expression of the multi-ligand receptor megalin LRP2 | Megalin^14^ | 4036 | [nsp10; nsp14; nsp7; nsp9; orf3a] | 1 |
|  | decreased expression of the multi-ligand receptor megalin LRP3 | SLC22A12^14^ | 116085 | [spike; nsp6; nsp7; orf9b; orf9c] | 2 |
|  | decreased expression of the multi-ligand receptor megalin LRP4 | SLC34A1^14^ | 6569 | [nsp11; nsp15; nsp7; orf9b] | 2 |
|  | decreased expression of the multi-ligand receptor megalin LRP5 | SLC34A3^14^ | 142680 |  | 2 |
|  | decreased expression of the multi-ligand receptor megalin LRP6 | SLC6A19^14^ | 340024 | [e; nsp10; nsp13; nsp14; nsp15; nsp4; nsp5; nsp6; nsp7; nsp9; orf10; orf3a; orf3b; orf8; orf9b] | 2 |
|  | amino acid transporter | EAAT3^14^ | 6505 | [spike; nsp6; nsp7; orf3b; orf9c] | 1 |
|  | amino acid transporter | PAT2^14^ | 153201 | [n; spike; nsp1; nsp11; nsp12; nsp13; nsp14; nsp15; nsp6; nsp7; nsp8; nsp9; orf10; orf3a; orf3b; orf7a; orf9b] | 2 |
|  | amino acid transporter | SIT1^14^ | 54716 |  | 2 |
|  | amino acid transporter | B^0AT1^14^ | 340024 |  | 2 |
|  | amino acid transporter | B^0AT3^14^ | 348932 |  | 1 |
|  | amino acid transporter | ASCT2^14^ | 6510 | [spike; nsp6; nsp7; orf3a; orf3b; orf8] | 2 |
|  | amino acid transporter | SNAT3^14^ | 10991 | [orf6; orf9c] | 2 |
|  | amino acid transporter | TAT1^14^ | 117247 | [nsp14; nsp4; orf6; orf9c] | 2 |
|  | amino acid transporter | TMEM27^14^ | 57393 |  | 2 |
|  | amino acid transporter | SLC7A9^14^ | 11136 |  | 2 |
|  | amino acid transporter | SLC3A1^14^ | 6519 | [nsp7] | 2 |
|  | impaired tubular handling of uric acid | URAT1^14^ | 116085 |  | 2 |
|  | impaired tubular handling of uric acid | OAT1^14^ | 9356 | [nsp4; orf9c] | 2 |
|  | impaired tubular handling of uric acid | OAT3^14^ | 9376 |  | 2 |
|  | impaired tubular handling of uric acid | OAT4^14^ | 55867 | [n; orf9c] | 2 |
|  | impaired tubular handling of uric acid | GLUT9^14^ | 56606 | [m; spike; nsp11; nsp15; nsp2; nsp6; nsp7; orf3b; orf8; orf9c] | 1 |
|  | impaired tubular handling of uric acid | ABCG2^14^ | 9429 | [spike; nsp6; nsp7; orf3a; orf3b; orf9b] | 2 |
|  | impaired tubular handling of uric acid | NPT1^14^ | 6568 |  | 1 |
|  | impaired tubular handling of uric acid | NPT4^14^ | 10786 |  | 2 |
| Enterocytes (gut organoids) | Defense response to virus | IFI6^15^ | 2537 |  | 2 |
|  |  | IFI27^15^ | 3429 |  | 2 |
|  |  | IFITM1^15^ | 8519 |  | 2 |
|  |  | MX1^15^ | 4599 | [orf9c] | 1 |
|  |  | RN7SK^15^ | 125050 | [e; nsp8] | 1 |
|  |  | ARC^15^ | 23237 | [nsp12] | 2 |
|  |  | DKK1^15^ | 22943 | [nsp4; orf8; orf9c] | 2 |
|  |  | MT1G^15^ | 4495 | [nsp4] | 2 |
|  |  | SAMD9^15^ | 54809 | [nsp15; nsp5_c145a] | 2 |
|  |  | IFIT3^15^ | 3437 | [n; nsp15] | 1 |
|  |  | C10orf99^15^ | 387695 |  | 1 |
|  |  | IFIT2^15^ | 3433 | [n] | 1 |
|  |  | NKX3-1^15^ | 4824 |  | 1 |
|  |  | LINC00941^15^ | 1E+08 |  |  |
|  |  | HSPA6^15^ | 3310 |  | 1 |
|  |  | IFIT1^15^ | 3434 | [n; nsp10; nsp15] | 1 |
|  |  | IFI44L^15^ | 10964 | [orf8; orf9b] | 2 |
|  |  | CMPK2^15^ | 129607 |  | 1 |
|  |  | RSAD2^15^ | 91543 | [nsp1; nsp11; nsp13; orf9b] | 2 |
|  |  | ISG15^15^ | 9636 | [m; nsp10; nsp11; nsp12; nsp6; nsp7; nsp8] | 2 |
|  |  | OAS2^15^ | 4939 |  | 2 |
|  |  | CXCL11^15^ | 6373 | [nsp4] | 2 |
|  |  | IFI44^15^ | 10561 | [nsp11; nsp4; nsp5_c145a; orf3b; orf8] | 1 |
|  |  | CXCL10^15,16^ | 3627 | [nsp4] | 2 |
|  |  | SLC34A2^15^ | 10568 | [m; nsp6] | 2 |
|  |  | IFNλ^16,17^ | 3458 | [spike; nsp11; nsp13; nsp4; nsp7; orf9c] | 3 |
|  |  | IFN b1^17^ | 3456 | [nsp10] | 2 |
|  |  | IFNL2^16^ | 282616 |  | 2 |
|  |  | IFNL3^16^ | 28617 |  | 1 |
|  |  | CCR1^16^ | 1230 | [spike; nsp15; nsp4; orf9c] | 2 |
|  |  | CCR8^16^ | 1237 | [nsp10; nsp2; orf9c] | 2 |
|  |  | IL16^16^ | 3603 |  | 2 |
|  |  | IL3^16^ | 3562 | [nsp10; nsp11; nsp7; orf8; orf9b] | 2 |
|  |  | CCR2^16^ | 729230 | [orf6; orf7a] | 2 |
|  |  | CCR5^16^ | 1234 | [nsp4; orf3a] | 2 |
|  |  | IL5^16^ | 3567 | [spike; nsp10; orf8] | 2 |

**Supplementary File 1B. Significant target for each viral protein.**

| **SARS-Cov-2 Proteins** | **Human proteins which are most effect** | **Number of proteins** |
| --- | --- | --- |
| **e** | [RN7SK; SLC6A19; MYL9; CKM; MYLK; MYH8; NEB; IL-33; JUN; CD80; ACE2; AREG; TGFB2; STXBP5] | 14 |
| m | [ISG15; SLC34A2; GLUT9; ATP2A2; ACTB; MYO3B; MYO7B; IL-2; IL-13; IL-4; LDHA; PGD; ATP1A1*; UQCRB; SQLE*; DGAT1; JUN; HMOX2*; SOD2; CD80; IL-7; PD-1; CD66b; CD68; CTSB; RRAS; CTSD; CCL3] | 28 |
| n | [IFIT3; IFIT2; IFIT1; PAT2; OAT4; MYL4; ACAT2; JUN; EIF2AK2; Ki67; CD163; UNC13A; Tau] | 13 |
| S | [IFNλ; CCR1; IL-5; SLC22A12; EAAT3; PAT2; ASCT2; GLUT9; ABCG2; ACTB; TPM1; TMOD1; MYH2; IL-2; IL-13; IL-4; IL-10; IL-17; IL-22; UQCRB; ACAA1; DGAT1; CD80; PD-1; CD66b; NOG; TNFSF10; NRG1; CCL4; STEAP3; CCL3L1; CCL7; SNAP25; STXBP5] | 34 |
| nsp1 | [RSAD2; PAT2; ACTC1; NEB; MYLK2; HK2*; CDK1*; JUN; FOXO1*; GDF11; IGFBP3; CCNB1; RRM2; CCNB2; GRN; RAB3A; Tau] | 17 |
| nsp10 | [IFIT1; ISG15; IFN b1; CCR8; IL-3; IL-5; Megalin; SLC6A19; MYL4; TNNI3; TNNT3; MYO3B; IL-13; IL-33; IL-4; PGLS; IDH1*; ACAT2; MVD; JUN; ERN1; CD80; CD86; CTLA-4; CD66b; ACE2; FCGR3Acd16; TIMP1; CCNB2; GTSE1; RAB3A; SNAP25; STXBP1; Tau] | 34 |
| nsp11 | [RSAD2; ISG15; IFI44; IFNλ; IL-3; SLC34A1; PAT2; GLUT9; ENO3; TPM4; CALM3; TPM1; CALM2; MYL6; MYH11; TPM3; TNNI3; CAPZB; IL-2; IL-6; IL-12; IL-13; HK2*; GPI; PFKP; ENO2*; PKM*; LDHA; ALDOA; PGK1; PGD; PGLS; RBKS; ATP1A1*; UQCRB; IDH1*; ACAA1; ACAT2; MVD; FOXO1*; SOD2; ERN1; DNAJB9; CD80; FAS; ITGAM  cd1b; TNFSF10; NRG1; C5; CTSB; CTSD; CCNB1; CCNB2; GRN; RAB3A; SNAP25; SNCA; STXBP1; Syp; Caspase 3] | 61 |
| nsp12 | [ARC; ISG15; PAT2; IKKβ; TNF-α; IL-2; IL-13; IL-17; ACAA1; JUN; ACE2; TNFSF1; AREG; GTSE1; Caspase 3] | 15 |
| nsp13 | [RSAD2; IFNλ; SLC6A19; PAT2; ENO3; CALM3; TPM1; CALM2; MYL6; TNNI3; TNNT3; MYO15A; IKKβ; IL-13; RBKS; HMGCS1*; NFKB1*; JUN; CCNB1; CCNB2; GTSE1; Tau; Caspase 3] | 23 |
| nsp14 | [Megalin; SLC6A19; PAT2; TAT1; MYH11; MYH6; IL-6; GPI; PFKP; ENO2*; LDHA; PGK1; PGD; IDH1*; ACAA1; ACAT2; BCKDK; MVD; FOXO1*; GCLC*; SOD2; TNFSF10'; CTSL; CTSD; BIRC5; IGFBP3; CCNB2; Caspase 3] | 28 |
| nsp15 | [SAMD9; IFIT3; IFIT1; CCR1; SLC34A1; SLC6A19; PAT2; GLUT9; MYL6; TNNI3; MYO1G; IL-12; PFKP; ENO2*; BPGM*; PGD; PGLS; RBKS; IDH1*; ACAA1; ACAT2; JUN; FOXO1*; GCLC*; SOD2; CD80; CTLA-4; PD-1; ACE2; TNFSF10; NRG1; BIRC5; IGFBP3; CCNB2; RAB3A; SNAP25; STXBP1; Caspase 3] | 38 |
| nsp2 | [CCR8; GLUT9; ATP2A2; MYO1G; IL-2; IL-13; IL-4; UQCRB; MVD; FOXO1*; HMOX2*; FAS; ACE2; IL-18; RRAS; CTSD; CCNB2; SNAP25; STXBP1; STXBP5] | 20 |
| nsp4 | [DKK1; MT1G; CXCL11; IFI44; CXCL10; IFNλ; CCR1; CCR5; SLC6A19; TAT1; OAT1; ENO3; MYLK2; IL-13; IL-4; ENO2*; LDHA; PGD; PGLS; RBKS; UQCRB; IDH1*; ACAT2; MVD; SQLE*; JUN; HMOX2*; ERN1; PD-1; ferritin h; FAS; ACE2; CXCL2; NRG1; C5; CCL4; CTSB; CTSD; IGFBP3; STEAP3; TGFB2; CCL3L1; CXCL6; CCL8; CCL2; CCL3; SNAP25; STXBP1; Caspase 3] | 49 |
| nsp5 | [SLC6A19; ACTB; MYH13; IL-6; IL-33; PKM*; NFKB1*; JUN; FOXO1*; Ki67; CD14] | 11 |
| nsp5_c145a | [SAMD9; IFI44; CAPZB; MYO3B; PFKP; ENO2*; ALDOA; PGK1; PGD; PGLS; UQCRB; IDH1*; ACAA1; ACAT2; SOD2; ERN1; GDF11; NOG; CTSB; CTSD; SNCA; Caspase 3] | 22 |
| nsp6 | [ISG15; SLC34A2; SLC22A12; SLC6A19; EAAT3; PAT2; ASCT2; GLUT9; ABCG2; ATP2A2; TPM4; TPM3; MYO1G; OBSCN; IL-6; IL-13; IL-4; HK2*; ATP1A1*; UQCRB; IFITM3*; ACAA1; ACAT2; BCKDK; FDFT1; SQLE*; DGAT1; HMOX2*; SOD2; ERN1; ACE2; NRG1; TIMP1; AREG; CTSB; CTSD; STEAP3; SNAP25; SNCA; STXBP5] | 40 |
| nsp7 | [ISG15; IFNλ; IL-3; Megalin; SLC22A12; SLC34A1; SLC6A19; EAAT3; PAT2; ASCT2; SLC3A1; GLUT9; ABCG2; ATP2A2; ACTB; CALM3; CALM2; MYO1G; MYH15; IL-2; IL-13; IL-4; ENO2*; PKM*; LDHA; DNAJC15*; ATP1A1*; UQCRB; FDFT1; MTTP; JUN; HMOX2*; CD80; CD86; CTLA-4; Ki67; FAS; CTSD; CCL; RAB27A; RAB3A; RIMS1; RPH3A; SNCA; Tau] | 45 |
| nsp8 | [RN7SK; ISG15; PAT2; IL-12; JUN; Ki67; Tau] | 7 |
| nsp9 | [Megalin; SLC6A19; PAT2; OBSCN; MYO1A; IL-6; PGD; JUN; GCLC*; ERN1; ATF4; CXCL2; TIMP1; TGFB2; CXCL6; CCL8; CCL2] | 17 |
| orf10 | [SLC6A19; PAT2; ACTB; MYL6; CKM; IL-2; IL-6; IL-4; ENO2*; LDHA; ATP1A1*; JUN; IL-7; ACE2; TNFSF10; CTSD; CXCL1; Tau] | 35 |
| orf3a | [CCR5; Megalin; SLC6A19; PAT2; ASCT2; ABCG2; TNNI3; TTN; OBSCN; IL-13; RBKS; FDFT1; DGAT1; ERN1; CD80; Ki67; FAS; NOG; TNFSF10; NRG1; CMTM2; STEAP3; SNAP25; STX1A; STXBP1; STXBP5; Caspase 3] | 27 |
| orf3b | [IFI44; SLC6A19; EAAT3; PAT2; ASCT2; GLUT9; ABCG2; ATP2A2; ENO3; ACTB; TPM4; TPM1; MYL6; MYH11; MYO1G; MYO7A; IL-3; GPI; LDHA; ALDOA; PGK1; PGD; ATP1A1*; UQCRB; IDH1*; CDK1*; IFITM3*; ACAA1; ACAT2; MVD; FDFT1; JUN; HMOX2*; SOD2; Ki67; SNCA] | 36 |
| orf6 | [CCR2; SNAT3; TAT1; ATP2A2; ENO3; CKM; TPM3; TMOD1; HK2*; ALDOA; PGK1; PGD; ATP1A1*; UQCRB; IDH1*; ACAT2; JUN; SOD2; CD80; PD-1; FAS; CD68; NRG1; CTSD; RRM2; TGFB2; UNC13A; RAB3A] | 28 |
| orf7a | [CCR2; PAT2; ATP2A2; MYLK2; IL-13; PFKP; PKM*; ACAT2; BCKDK; JUN; DNAJB9; CD80; Ki67; FAS; ACE2; CD68; NRG1; IL-18; STEAP3; Tau] | 20 |
| orf8 | [DKK1; IFI44L; IFI44; IL-3; IL-5; SLC6A19; ASCT2; GLUT9; MYH15; MYO1F; TNF-α; IL-1β; IL-2; IL-6; IL-12; IL-13; IL-33; IL-4; IL-10; IL-17; HK2*; MTTP; SOD2; ERN1; CD80; CRP; ACE2; MRC1 cd206; GDF11; NOG; NRG1; TIMP1; CTSD; IGFBP3; CCNB2; TGFB2; CCL7; SNCA] | 38 |
| orf9b | [IFI44L; RSAD2; IL-3; SLC22A12; SLC34A1; SLC6A19; PAT2; ABCG2; ENO3; TNNC1; CKM; TNNI3; CAPZB; IKKβ; IL-6; IL-33; IL-4; HK2*; PKM*; PGD; ACAT2; MVD; JUN; GCLC*; ERN1; CD80; CTLA-4; ACE2; TNFSF10; CTSB; DDIT4; IGFBP3; CCNB2; TGFB2; RIMS1; SNAP25; STXBP5; Tau] | 38 |
| orf9c | [MX1; DKK1; IFNλ; CCR1; CCR8; SLC22A12; EAAT3; SNAT3; TAT1; OAT1; OAT4; GLUT9; ATP2A2; ACTB; MYH6; MYH15; IL-2; IL-13; IL-4; IL-10; IL-17; IL-22; SQLE*; DGAT1; HMOX2*'; ERN1; DNAJB9; CD80; IL-7; PD-1; FAS; ACE2; NOG; NRG1; C5; CCL4; CMTM2; CCL3; CXCL9; RAB3A; STXBP5] | 41 |

**References:**

1. Zhou, R et al., Acute SARS-CoV-2 Infection Impairs Dendritic Cell and T Cell Responses, *Immunity*, **53**, 864-877.e5 (2020)
2. Akbari, A., Rezaie, J., Potential therapeutic application of mesenchymal stem cell-derived exosomes in SARS-CoV-2 pneumonia, *Stem Cell Res. Ther. Lond* **11**, 1–10 (2020)
3. Schub, D., et al., High levels of SARS-CoV-2 specific T-cells with restricted functionality in patients with severe course of COVID-19, *medRxiv*, 2020.07.08.20148718 (2020)
4. Wang, J., Jiang, M., Chen, X., Montaner, L. J., Cytokine storm and leukocyte changes in mild versus severe SARS‐CoV‐2 infection: Review of 3939 COVID‐19 patients in China and emerging pathogenesis and therapy concepts, *J. Leukoc. Biol*. (2020)
5. Jin, M., et al., Long-term Existence of SARS-CoV-2 in COVID-19 Patients: Host Immunity, Viral Virulence, and Transmissibility, preprint (2020)
6. Peruzzi, B. et al., Quantitative and qualitative alterations of circulating myeloid cells and plasmacytoid DC in SARS-CoV-2 infection, Immunology, **161**, 345-353 (2020)
7. Chen, Y., et al., The Novel Severe Acute Respiratory Syndrome Coronavirus 2 (SARS-CoV-2) Directly Decimates Human Spleens and Lymph Nodes, *medRxiv,* 2020.03.27.20045427 (2020)
8. Xiong, Y., et al., Transcriptomic characteristics of bronchoalveolar lavage fluid and peripheral blood mononuclear cells in COVID-19 patients, *Emerg. Microbes Infect*., **9**, 761-770 (2020)
9. Liao, M., et al., Single-cell landscape of bronchoalveolar immune cells in patients with COVID-19, *Nat. Med*., **26**, 6 (2020)
10. Ehrlich, A., Uhl, S., Ioannidis, K., et al., The SARS-CoV-2 Transcriptional Metabolic Signature in Lung Epithelium, *SSRN Electron. J*., 2020
11. Pérez-Bermejo, J. A., et al., SARS-CoV-2 infection of human iPSC-derived cardiac cells predicts novel cytopathic features in hearts of COVID-19 patients, *bioRxiv*, 2020.08.25.265561 (2020)
12. Liao, Y., et al., Distinct infection process of SARS-CoV-2 in human bronchial epithelial cell lines, *J. Med. Virol*., **92**, 2830-2838 (2020)
13. Ramani, A., et al., SARS-CoV-2 targets neurons of 3D human brain organoids, *EMBO J*., **39**,e106230 (2020)
14. Werion, A., et al., SARS-CoV-2 causes a specific dysfunction of the kidney proximal tubule, *Kidney Int.* (2020)
15. Lamers, M. M., et al., SARS-CoV-2 productively infects human gut enterocytes, *Science*, **369**, 50-54 (2020)
16. Zhou, J., et al., Infection of bat and human intestinal organoids by SARS-CoV-2, *Nat. Med*., **26**, 7 (2020)
17. Stanifer, M. L., et al., Critical Role of Type III Interferon in Controlling SARS-CoV-2 Infection in Human Intestinal Epithelial Cells, *Cell Rep*., **32**, 107863 (2020)
